# Identification of subtypes of anticancer peptides based on sequential features and physicochemical properties

**DOI:** 10.1038/s41598-021-93124-9

**Published:** 2021-06-30

**Authors:** Kai-Yao Huang, Yi-Jhan Tseng, Hui-Ju Kao, Chia-Hung Chen, Hsiao-Hsiang Yang, Shun-Long Weng

**Affiliations:** 1 Department of Medical Research, Hsinchu Mackay Memorial Hospital, Hsinchu City, 300 Taiwan; 2 Department of Medicine, Mackay Medical College, New Taipei City, 252 Taiwan; 3 Department of Obstetrics and Gynecology, Hsinchu Mackay Memorial Hospital, Hsinchu City, 300 Taiwan; 4 Mackay Junior College of Medicine, Medicine, Nursing and Management College, Taipei City, 112 Taiwan

**Keywords:** Cancer, Computational biology and bioinformatics, Drug discovery

## Abstract

Anticancer peptides (ACPs) are a kind of bioactive peptides which could be used as a novel type of anticancer drug that has several advantages over chemistry-based drug, including high specificity, strong tumor penetration capacity, and low toxicity to normal cells. As the number of experimentally verified bioactive peptides has increased significantly, various of in silico approaches are imperative for investigating the characteristics of ACPs. However, the lack of methods for investigating the differences in physicochemical properties of ACPs. In this study, we compared the N- and C-terminal amino acid composition for each peptide, there are three major subtypes of ACPs that are defined based on the distribution of positively charged residues. For the first time, we were motivated to develop a two-step machine learning model for identification of the subtypes of ACPs, which classify the input data into the corresponding group before applying the classifier. Further, to improve the predictive power, the hybrid feature sets were considered for prediction. Evaluation by five-fold cross-validation showed that the two-step model trained with sequence-based features and physicochemical properties was most effective in discriminating between ACPs and non-ACPs. The two-step model trained with the hybrid features performed well, with a sensitivity of 86.75%, a specificity of 85.75%, an accuracy of 86.08%, and a Matthews Correlation Coefficient value of 0.703. Furthermore, the model also consistently provides the effective performance in independent testing set, with sensitivity of 77.6%, specificity of 94.74%, accuracy of 88.99% and the MCC value reached 0.75. Finally, the two-step model has been implemented as a web-based tool, namely iDACP, which is now freely available at http://mer.hc.mmh.org.tw/iDACP/.

## Introduction

Cancer remains a significant health problem worldwide in the 2020s. WHO has estimated that the global cancer incidence would be over 23 million new cases per year by 2030. However, despite an increase in the number of therapeutics available, cancer treatments are often accompanied by significant adverse effects on healthy cells, including radiotherapy, surgery, and chemotherapy. Furthermore, cancer cells can cause dynamic altering in the genome, and capable of developing resistance to chemotherapeutic drugs^1^. Recently, several studies reported that the chemotherapeutic drug resistance results in a poor efficacy of non-specific anticancer drugs and high mortality rates^2,3^.While few studies have focused on identification of the mechanisms of cancer drug resistance, and developing novel drugs to combat them^4^. But the development of anticancer therapy with reduced drug resistance and side-effects remains a major challenge in this field. In recent years, increasing peptides have been discovered to have several bioactivities of medicinal interest, such as cancer treatment, diabetes management, and cardiovascular diseases control; the application of peptides in a variety of other therapeutic areas is growing rapidly. Anticancer peptides (ACPs) are a kind of bioactive peptides which exhibits antitumor activity, which are usually made up of 10 to 100 amino acids residues. ACPs are structurally similar to the antimicrobial peptides (AMPs), which are categorized as forming either an α-helix or β-sheet structures due to its failure to fold into a well-defined structure in olution^5^. ACPs could be used as a novel type of anticancer drug that has several advantages over chemistry-based drug, including high specificity, strong tumor penetration capacity, and low toxicity to normal cells. The preclinical and clinical trials in the use of peptide-based vaccines as anticancer are growing in recent years^6^.

Several studies have already shown that ACPs display a variety of mechanisms of action. It well-known that cancer cells can secrete a large amount of lactate anions across plasma membranes, leading to a significant density of negative charges on the surfaces of cancer cells^7^. Coincidentally, investigations have been revealed that ACPs contain high hydrophobicity and a positive net charge, selectively killing cancer cells by interacting with anionic cell membrane components of cancer cells^8^. While some of ACPs activates mitochondrial apoptosis pathway to mediate cancer cell death, such as lactoferricin B and different β-ACPs^9^. Except for activity at the membrane level, ACPs were also found to interact with essential proteins to inhibit angiogenesis, and recruit immune cells to kill cancer cells such as HNP-1^10^. However, the process of novel cancer drug development is usually labor intensive, time- and cost-consuming; thus, several in silico approaches have been developed for identification of potential bioactive peptides as therapeutic agents. Tyagi et al. developed a support vector machine (SVM) model trained with amino acid composition and binary profiles^11^. Hajisharifi et al. found that integrating Chou's pseudo-amino acid composition (PseAAC) and local alignment kernel could improve the prediction of ACPs^12^. Recently, several models have been proposed based on SVM and random forest (RF) algorithms by incorporating the multiple sequence features information, including amino acid composition, dipeptide composition, g-gap dipeptide compositions, atomic composition and physicochemical properties^13,14^. To improve the predictive power, recent studies have focused on selecting the important sequence-based features using the F-score^15,16^.

Although several computational approaches have been developed to discriminate the peptides with and without anticancer activities, these methods could not be used for the investigation of the differences in physicochemical properties of ACPs. With this, we were motivated to develop a method for identification of the subtypes of ACP based on sequence-based features and physicochemical properties. In this study, all of the experimentally validated ACPs were collected from the published databases. There are some databases which provide amino acid sequences and related literatures of ACPs, including APD3 (Release 2016-01, http://aps.unmc.edu/AP/)^17^, CancerPPD (Release 2015-01, http://crdd.osdd.net/raghava/cancerppd/)^18^ and SATPdb (Release 2016-01, http://crdd.osdd.net/raghava/satpdb/)^19^. To understand the comprehensive properties of ACPs, we provide a full characterization based on various features, including amino acid composition (AAC), dipeptide amino acid composition (DPC), composition of *k*-spaced amino acid pairs (CKSAAP) and physicochemical properties (PCP). Due to the difficulty of observing the conserved motifs from a large-scale sequence dataset, the training dataset has been divided into groups that share similar characteristics. Subsequently, each group is then built as a predictive model with above features using support vector machine (SVM) algorithms, and the predictive performance of each model was evaluated by fivefold cross-validation. Furthermore, an additional ACP dataset was divided from the raw dataset which completely blind to the training dataset, and an independent testing of state-of-the-art methods was performed on these data. To facilitate the study of anticancer peptides, we are motivated to develop a web tool for discriminating between ACPs and non-ACPs.

## Results

### Preprocessing of training and testing dataset

**Figure 1:**
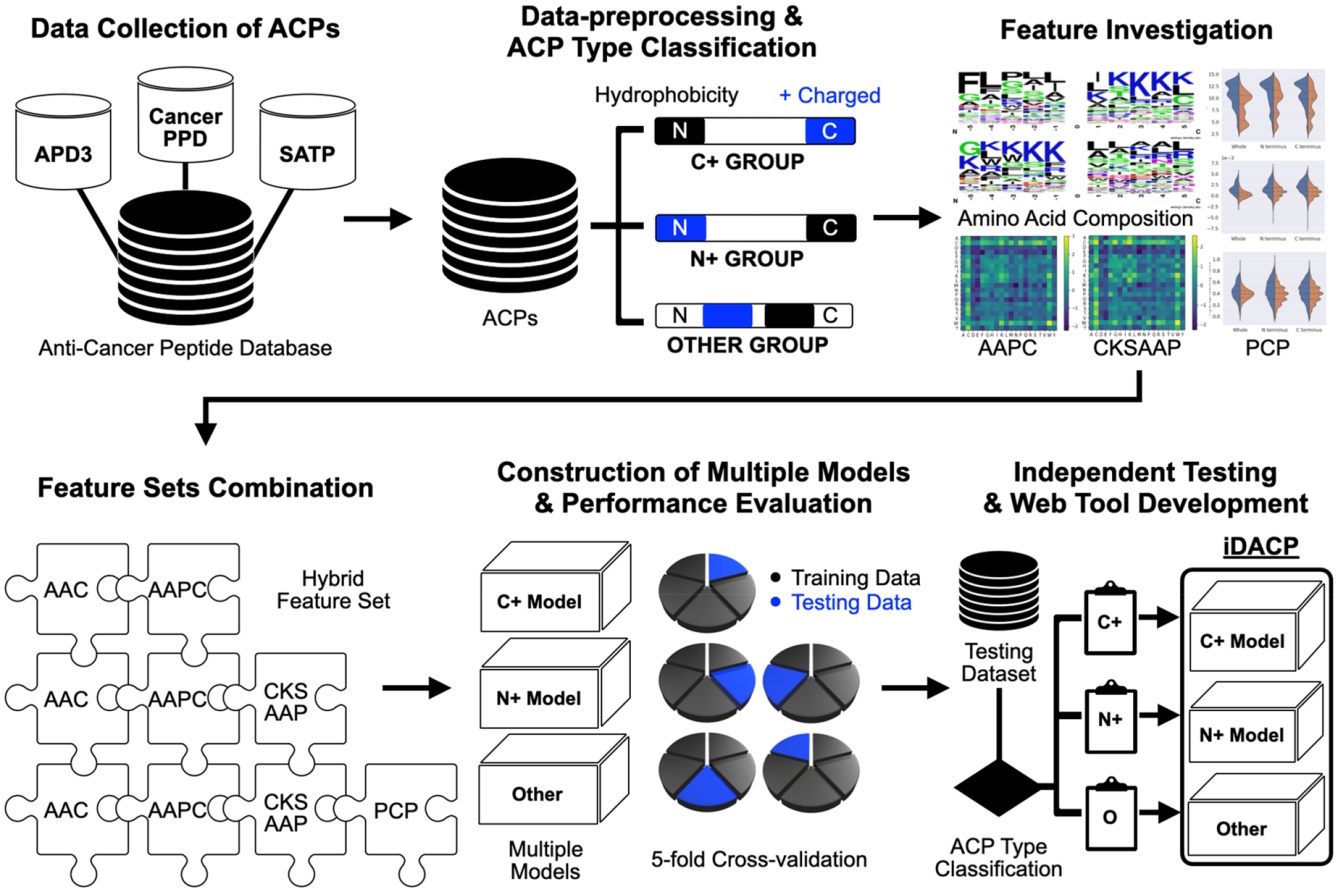
Analytical flowchart of iDACP including data collection, data preprocessing and type grouping, features investigation, feature sets combination, model construction and evaluation, and independent testing.

A detailed flow chart of the proposed method is shown in Fig. 1. With reference to the previous works, in this study, the peptides are composed more than 10 natural amino acid residues which were kept for further processing. In order to elude the overestimation of predictive performance, CD-HIT software package^20^ was used to remove homologous sequences from the training dataset with 90% sequence identity. In addition, the cd-hit-2d program was further applied across positive and negative training dataset with 100% sequence identity to avoid the false prediction. The non-homologous dataset consisted of 992 positive sequences and 1980 negative sequences, and the dataset was divided into training dataset and independent dataset.

Considering the limited ACP data availability, the non-ACPs were randomly extracted from the corresponding original dataset with the ratio of 1:2 between the number of positive and negative sequences. As shown in Table 1, of which 800 ACPs and 1600 non-ACPs were randomly selected for training dataset, then 192 ACPs and 380 non-ACPs for independent testing dataset.

**Table 1:** Data statistics of training and testing datasets after the removal of homologous sequences using CD-HIT program.

Sequence identity cut-off	Number of ACPs	Number of non-ACPs
Raw data	1354	2250
Sequence length > 10aa	1256	2250
Sequence identity < 90%	992	1980
Training dataset	800	1600
Independent testing dataset	192	380

*aa* amino acid, *ACPs* anti-cancer peptides, *non-ACPs* non-anti-cancer peptides.

### Composition of amino acids in the ACPs

**Figure 2:**
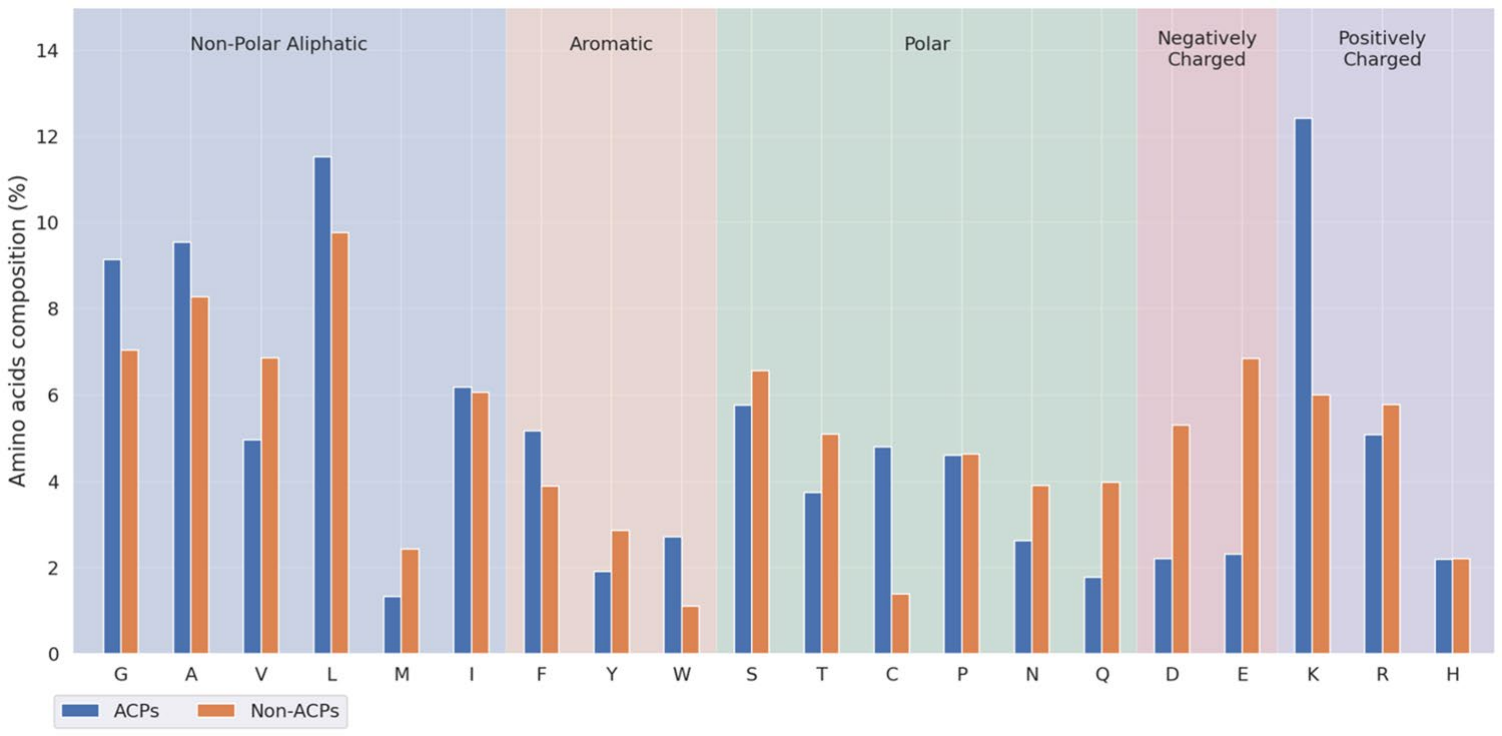
Investigation of composition of twenty amino acids of ACPs and non-ACPs.

The comparison of composition of amino acids between ACPs and non-ACPs was performed as shown in Fig. 2, which represents the enrichment of G (Gly, glycine), A, L, F, W and K residues in ACPs. The dominance of these amino acid residues indicates its contribution in peptide-membrane interactions. Figure 2 indicates that, the positively charged residue K occurs at a highest frequency in the peptides with anticancer activities; on the contrary, the negatively charged residues D (Asp, aspartate) and E (Glu, glutamate) which residues have a much lower frequency.

**Figure 3:**
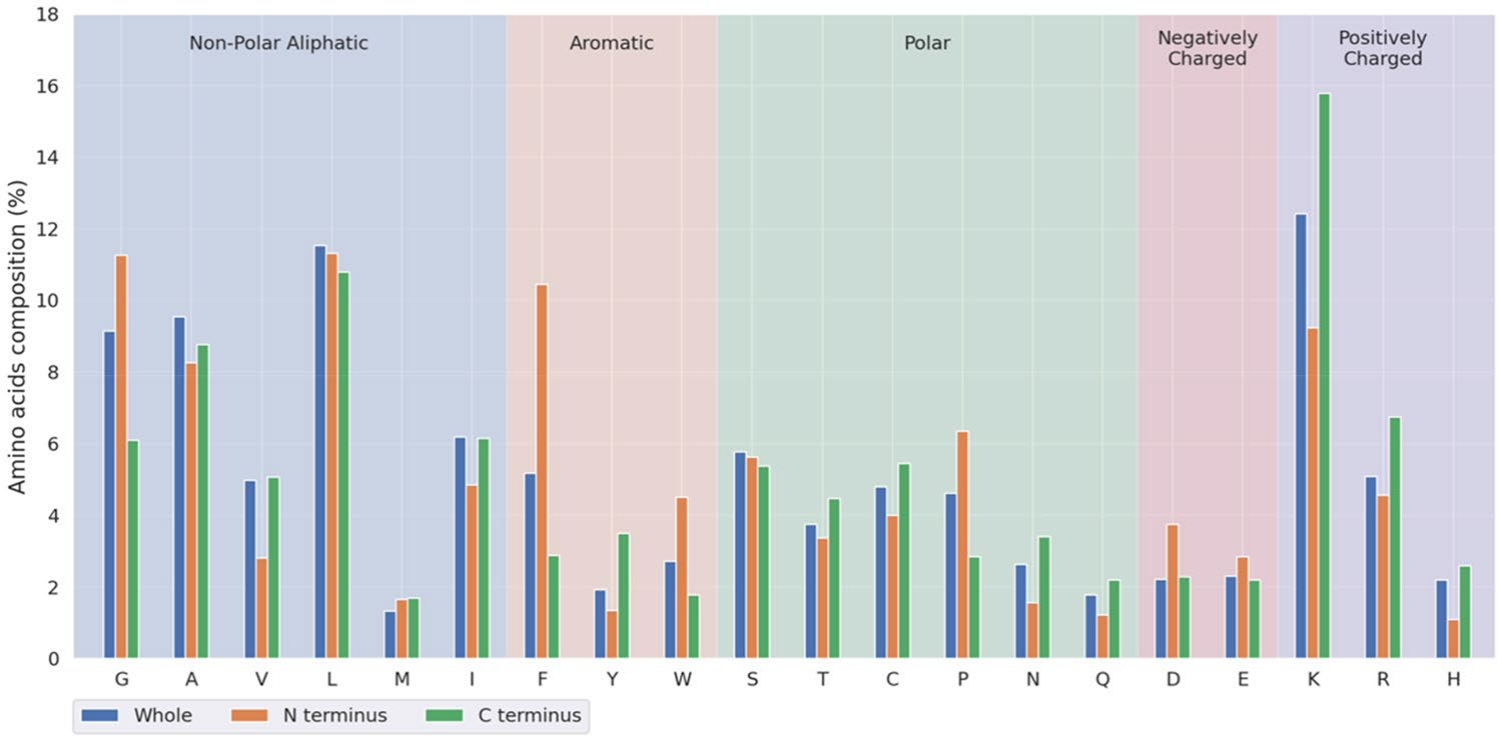
Investigation of composition of twenty amino acids between the N- and C-terminal regions of ACPs.

Furthermore, we performed a measurement of the amino acid composition at the N- and C-terminus of peptides based on the training dataset. As shown in Fig. 3, the hydrophobic non-polar aliphatic residue (G) and the aromatic residue (F/W) are over-represented at the N-terminus of ACPs. In contrast to the N-terminus of ACPs, a remarkable enrichment of the positively charged residue (K/R/H) is observed in C-terminus. Likewise, the results indicated that the positively charged region could serve as a good indicator to determine whether or not the peptide present the ability to inhibit or suppress the cancer progression, and the similar results were also observed in other comparative studies^11,14^.

**Figure 4:**
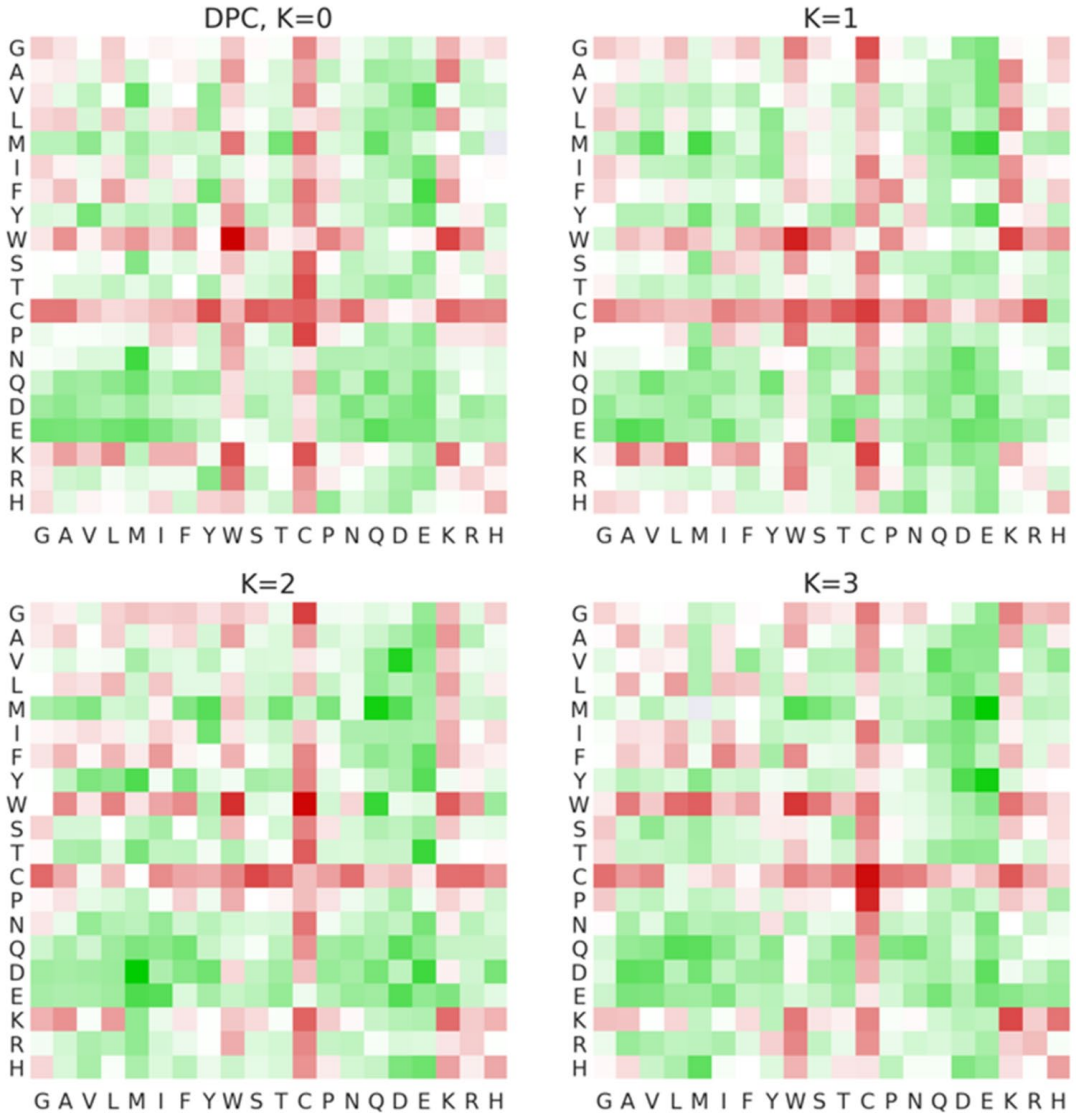
The frequency differences of 20 × 20 amino acid pairs between ACPs and non-ACPs.

In an attempt to compare of the composition of amino acid pairs between ACPs and non-ACPs, the composition of *k*-spaced amino acid pairs was also applied for investigation of ACPs in this study. The encoding scheme is based on the frequency of amino acid pairs that are separated by *k* number of residues (*k* = 0, 1, 2, 3), for instance, CK, CxK, CxxK and CxxxK. Figure 4 shows the frequency differences of 400 k-spaced amino acid pairs in the 20 × 20 matrices, the elements of the matrix marked in red indicates that the overrepresentation of the amino acid pairs in ACPs, while green means the under-representation. The amino acid composition of dipeptides was taken into account when *k* = 0, this investigation shows that WW, WK, PC, TC, CY, KW, KC, CS, CC and SC are over-represented in ACPs. When *k* = 1, it would be noticed that C residues paired with other residues are overrepresented such as CxC, KxC, GxC, CxR, CxW, CxT and CxN as well as WxW, WxK and KxL pairs; ACPs contain remarkable enrichments of WxxW, GxxC, CxxS, WxxK, TxxC, KxxC, CxxT, CxxG and KxxK when *k* = 2, and CxxxC, PxxxC, WxxxW, KxxxK, CxxxK and WxxxM pairs are enriched when *k* = 3. Thus, a various number of hydrophobicity and positively charged amino acids in the ACP sequence were found which play a vital role in discriminating between ACP and non-ACPs.

**Figure 5:**
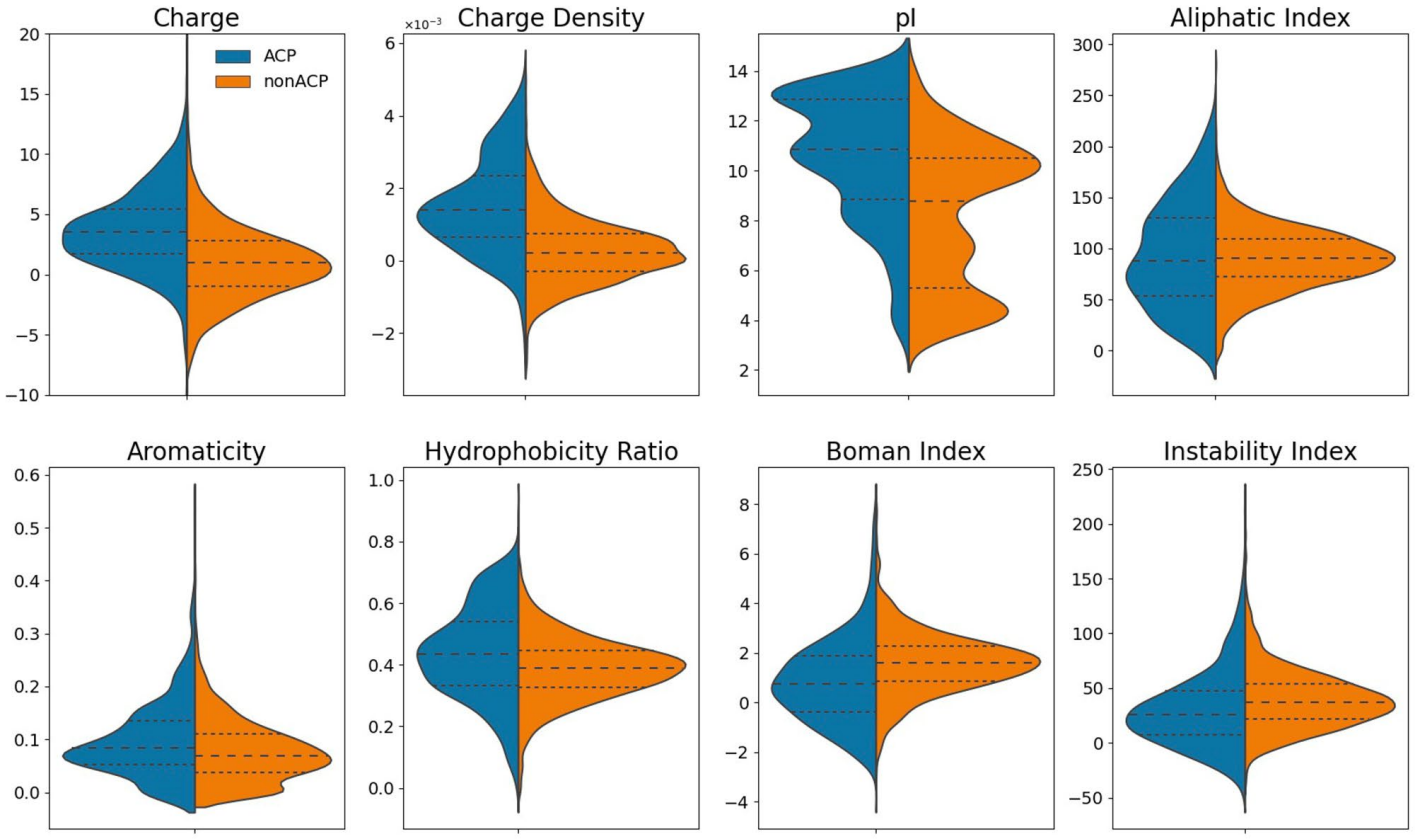
Comparison of the physicochemical property profiles between ACPs and non-ACPs.

The physicochemical properties were calculated for the whole peptide by using modlAMP tools, and the results of statistical analysis are shown in Fig. 5. More than 85% of ACPs containing a net positive charge of at least + 2 and with a high isoelectric point (pI) in the training data, especially in the C-terminus, which provide the evidence that ACPs is highly cationic at neutral pH, facilitating electrostatic interaction with the cancer cell membrane. Recent study^21^ has shown that the cell-penetrating ability play a critical role in the specific targeting of cationic peptides with anticancer activities to the cancer cells.

The comparison of the relative hydrophobicity between ACPs and non-ACPs indicated that the hydrophobicity ratio of ACPs was slightly higher than non-ACPs, the mean value of 0.44 and 0.39, respectively; as well as the mean of 0.097 and 0.078 in aromaticity ratio, respectively. The aliphatic index is described as the relative volume occupied by the aliphatic side chains, which correlates with the thermal stability of a protein molecule^22^. Referring to the statistical analysis, the mean values of aliphatic index for ACPs is 93.45 and for Non-ACPs is 90.03, since the *p*-value is greater than 0.05, we concluded that there is no statistically significant difference between the two groups. The instability index measures the stability of a protein in nature, if the index is greater than 40 then it is believed to be an unstable molecule^23^. The statistical result indicates that 800 ACPs in the training data of which about 67.5% were classified as stable, which are significantly more stable than non-ACPs. The Boman index estimates the potential for a protein to interact with other proteins, and a low index value (≤ 1) indicates the protein has a low side effect and toxicity profile^24^. Compared with the peptides without anticancer activities, a lower index value of ACPs reveals that their inability to interact with other proteins which may offer less side effects. Additionally, all of these physicochemical properties were also assessed for N- and C-terminus of the peptides; likewise, except for the properties related to electric charge, there is no significantly difference between two groups. The result shows that 49% of ACPs contains a higher cationic charge at the C-terminus compared to identical peptides with the N-terminus, and the hydrophobic residues are enriched in their N-terminal flanking.

### Cross-validation performance in the prediction of ACPs

In the binary classification between 800 ACPs and 1,600 non-ACPs, the SVM models trained with sequence-based features such as AAC, DPC, CKSAAP as well as PCP are evaluated the predictive performance by using five-fold cross-validation.

As presented in Table 2 (see more detail in Additional File 1: Table S1), the SVM model trained with AAC provides the best overall performance with a sensitivity of 86.23%, specificity of 87.25%, accuracy of 86.91%, balanced accuracy of 86.74% and gives the highest MCC value of 0.72 in classifying between ACPs and non-ACPs. The model trained with DPC yields a sensitivity of 85.38%, specificity of 84.63%, accuracy of 84.88%, balanced accuracy 85.0% and MCC of 0.68 on ACPs prediction. The models trained with CKSAAP could also provide an exceptional performance, no matter the value of *k*; the model trained with C1SAAP (composition of 1-spaced amino acid pair) could provide a performance with 85.98% sensitivity, 86.31% specificity and 86.14% balanced accuracy, the C2SAAP model provide a performance with 85.50% sensitivity, 86.34% specificity and 85.92% balanced accuracy, and the model provide a performance with 86.63% sensitivity, 86.55% specificity and 86.59% balanced accuracy when *k* = 3. In particular, the model trained with PCP gives a sensitivity of 71.53%, specificity of 71.05%, balanced accuracy of 71.29%, and MCC of 0.41. Comparing to the models trained with AAC, DPC and CKSAAP, the PCP model was constructed only using a small number of features, of which a total of 8 physicochemical properties were used merely as features for classification, and gives a passable performance. This preliminary analysis indicates that some of the properties might play an important role in identification of ACPs.

**Table 2:** Five-fold cross validation results of the models trained with single feature.

Feature	Sen. (%)	Spec. (%)	Acc. (%)	BAcc. (%)	MCC
AAC	86.23 ± 0.56	87.25 ± 0.28	86.91 ± 0.23	86.74 ± 0.28	0.72 ± 0.01
DPC	85.38 ± 0.36	84.63 ± 0.50	84.88 ± 0.35	85.00 ± 0.30	0.68 ± 0.01
CKSAAP, k = 1	85.98 ± 0.54	86.31 ± 0.46	86.20 ± 0.44	86.14 ± 0.45	0.70 ± 0.01
CKSAAP, k = 2	85.50 ± 0.27	86.34 ± 0.41	86.06 ± 0.26	85.92 ± 0.21	0.70 ± 0.00
CKSAAP, k = 3	86.63 ± 0.13	86.55 ± 0.26	86.58 ± 0.20	86.59 ± 0.17	0.71 ± 0.00
PCP	71.53 ± 0.95	71.05 ± 0.47	71.21 ± 0.60	71.29 ± 0.68	0.41 ± 0.01

*Sen.* Sensitivity, *Spec.* specificity, *Acc.* Accuracy, *BAcc.* balanced accuracy, *MCC* Matthews correlation coefficient. The values represent the mean and standard deviation of all measurements.

### Classification of the subtypes of ACPs based on the charge distribution

However, although the above models can provide good prediction results, an interesting problem about amino acid sequence arrangements should be noted here. Based on the sequence-based features such as AAC, DPC and CKSAAP, the investigations were performed for the whole sequence only but not for the specific regions of the peptide. For instance, given two protein sequences (a) FLWCPCLKKC and (b) CPCLKKCFLW, the pair of equal length segments that, both of sequences have the identical composition of amino acids (F: 10%, L: 20%, W: 10%, C: 30%, P: 10% and K: 20%). Nevertheless, the former sequence meets the characteristics of the peptides with anticancer activities that contains a hydrophobic region and a positively charged region flanking the N- and C-terminus of the peptide, respectively. Unlike the former one, the latter sequence possesses the same amino acid composition as that of the above, but it does not have any pattern of arrangement or otherwise. Although the present methods trained with the sequence-based features might appear to provide a good performance, but the above problem is persistent.

**Figure 6:**
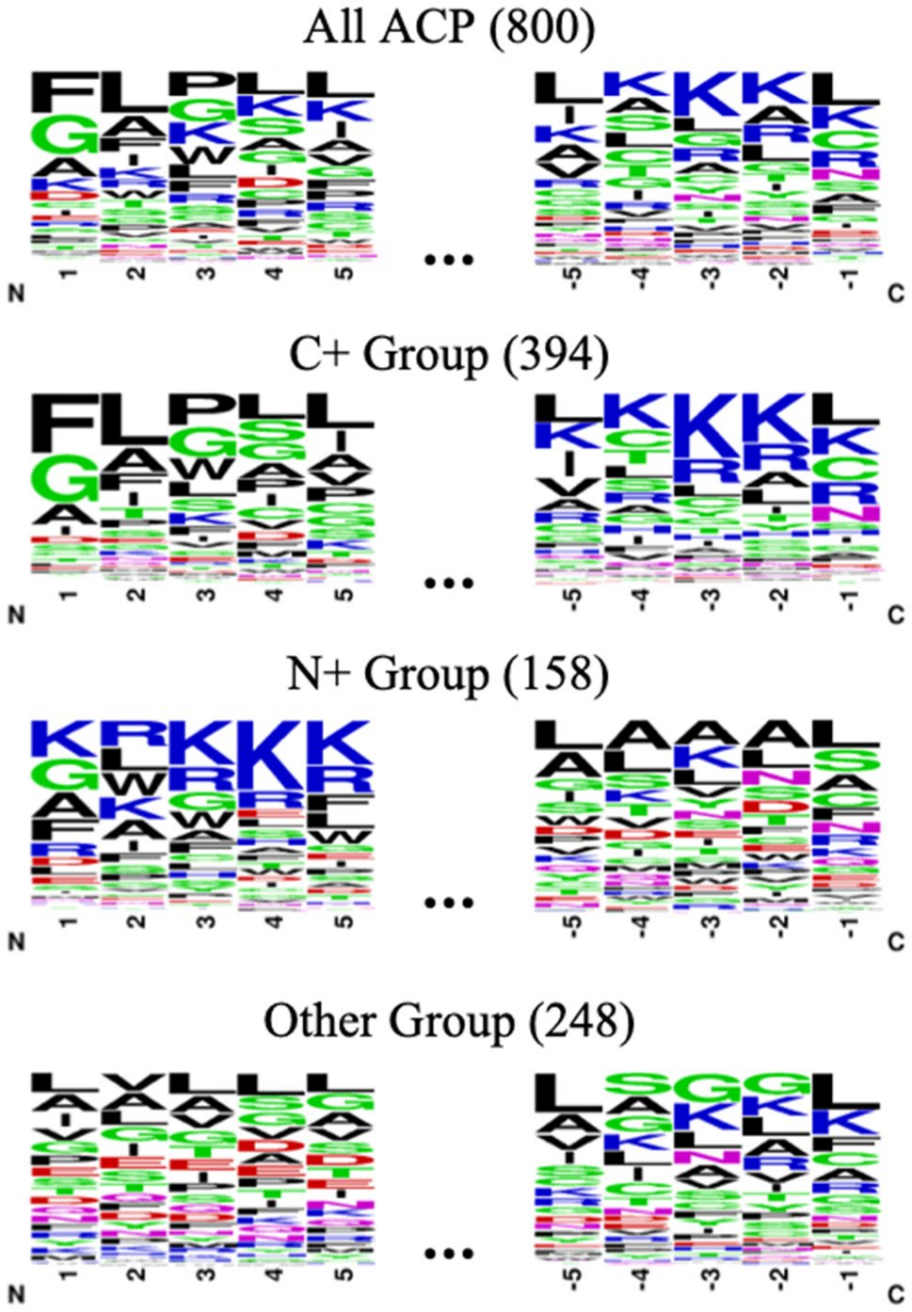
Position-specific amino acid composition of the N- and C-terminal regions in the different subtypes of ACPs.

Several studies have reported that ACPs contain a high proportion of hydrophobic residues at the N-terminus, while more positively charged residues were found at the C-terminus^14^, which is believed to play a crucial role in the binding and selective disruption of cancer cell membrane^25,26^. However, based on the amino acid sequence analysis, we found that many of the ACPs in our training dataset that have different conformations of amino acids. Therefore, we have carried an exhaustive analysis of the different regions of ACPs which might interact with cancer cell membranes. WebLogo^27^ was used to explore the position-specific composition of amino acids at the N- and C-terminus of ACPs and the frequency plots were visualized as shown in Fig. 6. However, there is no consensus sequence at the N- or C-terminus of the ACPs; thus, all of 800 ACPs were classified into different groups for further analysis based on the net charge distribution.

**Figure 7:**
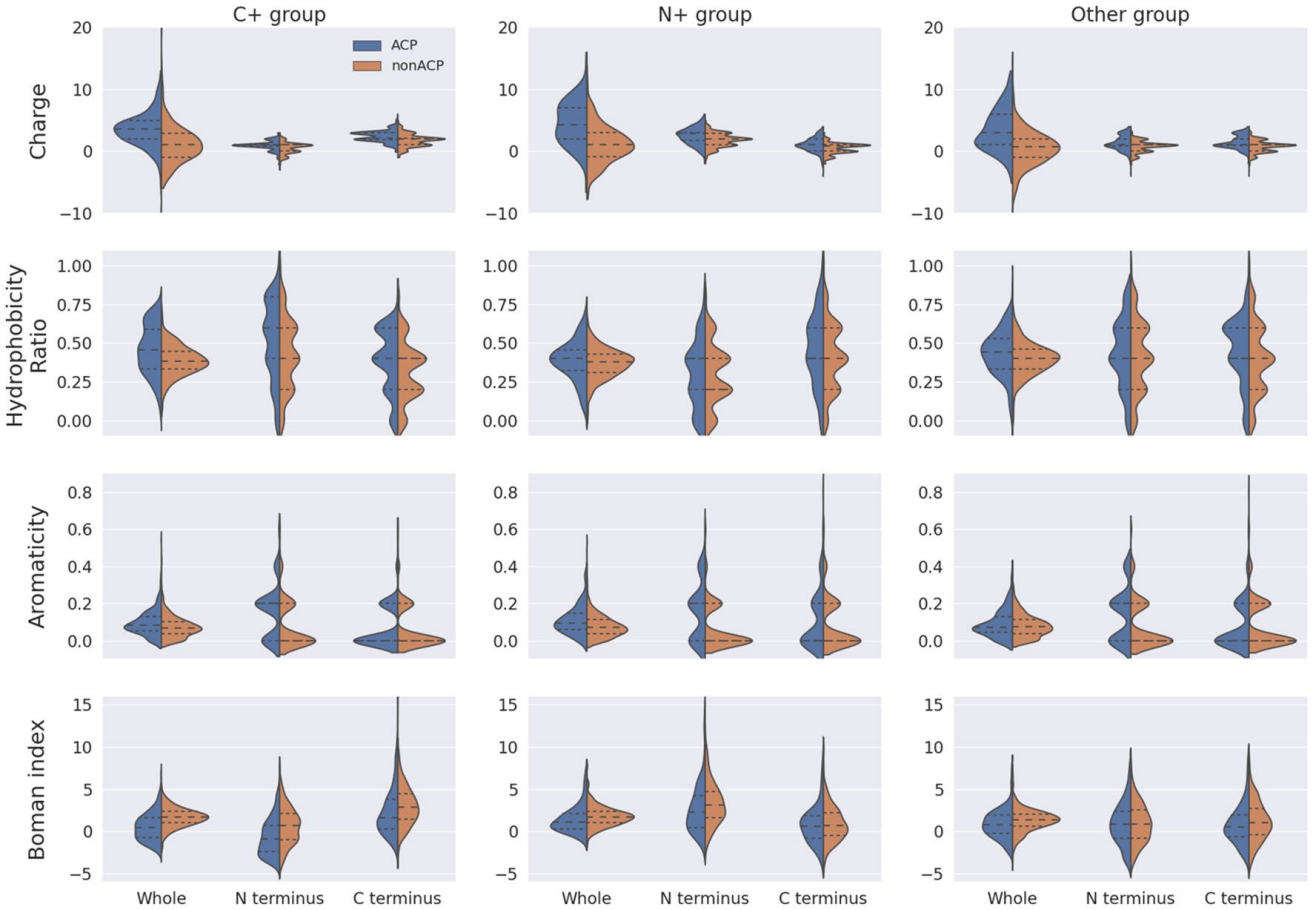
Physicochemical property profiles of N- and C-terminus in the different subtypes of ACPs.

According to the criteria described in the method section, training data are further classified as shown in Table 3, of which 394 positive data and 428 negative data are belong to the “group C+”, while 158 positive data and 508 negative data are classified into the “group N+”, and the remaining data (248 positive data and 664 negative data) into the “group Other”. When the subtypes of ACPs were further analyzed, it has been observed that the significant difference among three groups in various physicochemical properties, including net charge, hydrophobicity ratio, aromaticity and Boman index, as shown in Fig. 7.

**Table 3:** Data statistics for each type of ACP in the training and testing datasets.

Dataset	ACPs	Non-ACPs
Training dataset	800	1600
Group C+	394	428
Group N+	158	508
Group other	248	664
Testing dataset	192	380
Group C+	94	103
Group N+	39	131
Group other	59	146

### A two-step method for identification of the subtypes of ACP

Subsequently, the sequence investigation was also performed in the three groups. The ACPs belong to the group C+ which comprises positively charged residues at the C-terminal region and a stretch of hydrophobic amino acids at the N-terminal region. On the contrary, several ACPs of group N+ that preserve the positive charge at N-terminus and have a C-terminal hydrophobic region. The analytical results suggested that each subtype of ACPs has its own motif which might be associated with a distinct mechanism of killing cancer cells. Therefore, here we proposed a two-step method to identify the subtypes of ACPs. Given a peptide sequence, the net charges of the first and the least 5 amino acid residues were calculated, and the subtype was defined depending on which area contains a higher concentration of positively charged residues. Then, the peptide would be input to the corresponding model for prediction. As shown in Table 4 (see more detail in Additional File 1: Table S2), overall, the SVM models trained with AAC provided the best performance with a sensitivity of 85.45%, specificity of 84.09%, accuracy of 84.54%, balanced accuracy of 84.77%, and MCC value of 0.67 in discriminating between ACPs and non-ACPs.

**Table 4:** Five-fold cross validation results of the two-step models trained with the single feature.

Feature	Sen. (%)	Spec. (%)	Acc. (%)	BAcc. (%)	MCC
AAC	85.45 ± 0.74	84.09 ± 0.78	84.54 ± 0.41	84.77 ± 0.32	0.67 ± 0.01
DPC	83.83 ± 0.60	80.24 ± 0.95	81.43 ± 0.61	82.03 ± 0.50	0.61 ± 0.01
CKSAAP, k = 1	84.65 ± 0.92	82.33 ± 0.49	83.10 ± 0.40	83.49 ± 0.48	0.64 ± 0.01
CKSAAP, k = 2	83.95 ± 1.02	82.39 ± 0.44	82.91 ± 0.51	83.17 ± 0.61	0.64 ± 0.01
CKSAAP, k = 3	84.75 ± 0.72	82.68 ± 0.76	83.37 ± 0.45	83.71 ± 0.38	0.65 ± 0.01
PCP	73.55 ± 1.82	69.25 ± 1.99	70.68 ± 1.13	71.40 ± 0.93	0.41 ± 0.02

*Sen.* Sensitivity, *Spec.* specificity, *Acc.* Accuracy, *BAcc.* balanced accuracy, *MCC* Matthews correlation coefficient. The values represent the mean and standard deviation of all measurements.

### Cross-validation performance of the models trained with multiple types of features

In order to enhance the predictive capability, the combinations of different types of features were used to train the hybrid models for ACP prediction that were also evaluated by five-fold cross-validation. Comparing to the models trained with AAC feature is presented in Table 5 (see more detail in Additional File 1: Table S3), the SVM model trained with the combination of AAC and DPC features could slightly improve the performance with a sensitivity of 85.85%, a specificity of 85.45%, an accuracy of 85.58%, balanced accuracy of 85.65% and MCC of 0.69. According to the evaluation criteria, the model trained by combining AAC, DPC and PCP exhibited the best overall performance among various predictive models with a sensitivity of 86.05%, specificity of 85.78%, accuracy of 85.87%, balanced accuracy of 85.91%, and MCC value of 0.70. Additionally, the model trained by combining AAC, DPC, CKSAAP and PCP could provide a comparable performance to the models trained with AAC, the balanced accuracy value reached 85.41%. The comparison results indicated that all of the models trained with multiple types of features can provide better performance than single feature representation.

**Table 5:** Five-fold cross validation results of the two-step models trained with the hybrid feature sets.

Feature	Sen. (%)	Spec. (%)	Acc. (%)	BAcc. (%)	MCC
AAC + DPC	85.85 ± 0.66	85.45 ± 0.25	85.58 ± 0.36	85.65 ± 0.43	0.69 ± 0.01
AAC + DPC + PCP	86.05 ± 0.63	85.78 ± 0.25	85.87 ± 0.27	85.91 ± 0.34	0.70 ± 0.01
AAC + DPC + CKSAAP	86.18 ± 0.49	84.28 ± 0.29	84.91 ± 0.29	85.23 ± 0.33	0.68 ± 0.01
AAC + DPC + CKSAAP + PCP	86.03 ± 0.58	84.79 ± 0.27	85.20 ± 0.33	85.41 ± 0.38	0.68 ± 0.01

*Sen*. Sensitivity, *Spec.* specificity, *Acc.* Accuracy, *BAcc.* balanced accuracy, *MCC* Matthews correlation coefficient. The values represent the mean and standard deviation of all measurements.

### Performance evaluation by independent testing datasets

Overfitting is a modeling error which the model tends to perfectly fit the observed data but performs poorly on unseen data during training. In order to avoid overfitting, an additional dataset was divided from the non-homologous dataset which consisted of 192 ACPs and 380 non-ACPs, and then these data were used to verify the predictive performance of the proposed method. Following the two-step process as outlined above, the independent testing dataset consisted of 94 ACPs and 103 non-ACPs for the group C+, 39 and 131 for group N+, as well as 59 and 146 for group Other as shown in Table 3. In recent years, many tools have been developed to predict the peptides with anticancer activities based on sequential or structural features. However, all of the published prediction tools could not predict the different types of ACPs. To further demonstrate the effectiveness of our method, five prediction tools, ACPred (Release 2019, http://codes.bio/acpred/)^28^, ACPred-FL (Release 2018, http://server.malab.cn/ACPred-FL/)^29^, Anti-CP (v2.0, https://webs.iiitd.edu.in/raghava/anticp2/)^30^, iACP (Release 2016, http://lin.uestc.edu.cn/server/iACP)^13^ and mACPpred (Release 2019, http://thegleelab.org/mACPpred/)^16^ are available for the comparison of predictive performance based on independent testing datasets. Finally, the proposed method provides the best performance, with a sensitivity of 77.60%, a specificity of 94.74%, an accuracy of 88.99%, a balanced accuracy of 86.17%, and the MCC value reached 0.75 as presented in Table 6 (see more detail in Additional File 1: Table S4). In summary, the comparison result indicates that the proposed method can outperform other tools in overall and can handle class imbalance in classification between ACPs and non-ACPs.

**Table 6:** Comparison of independent testing results between our method and the available prediction tools.

Tools	Sensitivity (%)	Specificity (%)	Accuracy (%)	B. Accuracy (%)	MCC
iDACP	77.60	94.74	88.99	86.17	0.75
ACPred	75.97	84.21	81.84	80.09	0.58
ACPred-FL	57.79	25.79	35.02	41.79	− 0.16
Anti-CP	100	0.29	34.03	50.15	0.03
iACP	65.10	75.53	72.03	70.32	0.40
mACPpred	71.35	94.47	86.71	82.91	0.70

*B. Accuracy.* balanced accuracy, *MCC* Matthews correlation coefficient.

### Implementation of web-based tool for anticancer peptides

Developing the novel anticancer peptide drugs still encounter equipment and technical difficulties, including expensive, time-consuming and labor-intensive process. Therefore, an effective prediction method should be developed to identify potential peptides with anticancer activities. After the validation testing, a web-based online tool for automatic prediction of subtypes of ACPs was developed based on the two-step model trained with the hybrid features. Users can input the peptide sequences in FASTA format, the system automatically reports the prediction results, including the probability of prediction and the bar plot for amino acid composition of whole peptide. The present method is expected to be a helpful reference for the researchers working in the field of development of novel anticancer drugs.

## Discussion

This study contributes to providing a comprehensive characterization of ACPs based on analysis of sequence composition and physicochemical properties. In this study, the first challenge is the imbalanced dataset problem. Most traditional machine learning methods may be limited in their capacity to classify imbalanced datasets. However, in imbalanced data classification, it has been reported that SVM can give a higher accuracy in predictive modeling than other algorithms^31^. In SVM with the RBF kernel, two major parameters are used to optimize the training model; the parameter C controls the trade-off between classification of training instances accurately and a smooth decision boundary, and the parameter Gamma defines how far the influence of a single training instance reaches. Through adjusting the two parameters in the algorithm, the generalized performance can be controlled in high-dimension space, and minimizing the number of misclassified instances. Thus, due to the strong theoretical foundations, SVM algorithm was chosen to solve the problem of imbalanced data in this work. Additionally, balanced accuracy was used to evaluate the model performance in this work, which is a better metric that more appropriate for mining imbalanced datasets.

Aspects regarding the sequence-based analysis, we found that AAC plays an integral role in the structure and function of ACP. Previous works have exhibited the hydrophobic amino acids such as G, A, L and F were preferential residues at the N-terminus of ACPs, while V, C, L and the positively charged amino acid K were likely to be found at the C-terminus^11,13,14^. Notably, the other previous study has indicated that, the peptides composed of rich hydrophobic positively charged lysine and arginine that can select anionic membranes on cancer cells and disrupt the cell membrane through the snorkeling mechanism, which play an important role in cancer cell toxicity^32^. Moreover, the study by Ma et al. carried out that the fusion of hydrophobic and positively charged amino acids in a phage lysin can effectively kill E. coli through destroying the cell membrane, and the antimicrobial activity gradually increasing with the positive charge at the C-terminus of the peptides ^33^. Due to the membrane of cancerous cells also contains the negatively charged compound phosphatidylserine (PS) more than the normal cells, thus the over-representation of positively charged residue in ACPs is reasonable. With the increasing number of the positively charged residues, the peptides can easily bind to the negatively charged cancer cell membrane by the electrostatic interactions. ^34,35^.

In this study, there are three major subtypes of ACPs that are defined based on the distribution of positively charged residues, including group C+, group N+ and group Other. According to the comparative analysis of the N- and C-terminal amino acid composition among the groups, it reveals that one of the termini contains a high relatively of positively charged amino acids, while the presence of higher frequencies of hydrophobic amino acids at the opposite terminus. It should be further studied whether the different types of ACPs may correspond to different mechanisms of the anticancer activities. Additionally, the analysis of the physicochemical properties reveals that ACPs carry much higher charge density and hydrophobicity ratio than non-ACPs. The positive charged amino acids of the N- or C-terminus might enhance the electrostatic interaction between the positively charged peptide and the negatively charged cancer cell membrane. As stated previously, these investigations suggested that the composition of amino acids can play a crucial role in distinguishing between ACPs and non-ACPs.

According to the results of the comprehensive analysis, the model trained with the combination of AAC, DPC and PCP provides the best overall performance with a sensitivity of 86.05%, specificity of 85.78%, accuracy of 85.87%, balanced accuracy of 85.91%, and MCC value of 0.70, which was chosen as the final model for discriminating the peptides with or without anticancer activity. In order to objectively evaluate the performance of the proposed model, a comparison among published tools using the independent testing dataset is given. The proposed model provided the highest balanced accuracy of 86.17% compared to the other tools, with significant differences in balanced accuracy (3% to 45%).

## Conclusion

Ultimately, for the first time, we were motivated to develop a two-step machine learning model for identification of the subtypes of ACPs based on the sequenced-based features and physicochemical properties, which classified the input data into the corresponding group before applying the classifier. Otherwise, to facilitate the research and development of novel cancer drugs, iDACP, a reliable prediction tool for the identification of subtypes of ACPs has been developed, which is now freely available at http://mer.hc.mmh.org.tw/iDACP/.

Excitingly, in vivo experiments have been designed to support the in silico predictions, which we are currently performing to validate the anti-cancer activity of the predicted candidates. Besides, we plan to collect more data and conduct additional analyses in the future to identify the ACPs with specific toxicity against each type of cancer, and to improve the performance of the model that the feature selection algorithm will be implemented further to explore the crucial features.

## Methods

### Data collection

A total of 1,390 experimentally confirmed ACPs were collected from the published literatures^12,16,29,36^ and public databases, including APD3^17^, CancerPPD^18^ and SATPdb^19^. Although we had collected the experimentally validated ACPs from published databases and literatures as well as possible, but with only slightly more than a thousand records. Moreover, due to the lack of the experimental data for ACPs such as synthesis, structures, mechanisms, selective toxicity to cancer cells and effective concentration etc., which makes it difficult to further investigate this matter using in silico method. In addition, AntiCP^11^ is a public web tool allows the users to predict as to whether a query sequence is likely to be ACP. As the authors stated, since the lack of experimentally validated non-ACPs, thus a total of 2,250 unique peptides were randomly extracted from reviewed proteins in Swiss-Prot database which having length ranging from 10 to 55 amino acids, and the proteins cannot be annotated to anticancer activity or related terms, including anti-cancer, anti-tumor, apoptosis, and programmed cell death. These datasets were considered as the positive and negative data, respectively, which were used for further analysis in this study.

### Features investigation and encoding

This study focused on the investigation of sequence-based characteristics of experimentally confirmed ACPs, and each peptide sequence should be transformed into a numeric vector based on the above features to construct a supervised learning model

#### Amino acid composition (AAC)

The AAC describes the frequencies of 20 types of native amino acids in a given protein sequence^37^. Given a peptide sequence*,* the 20 elements represent the number of occurrences of 20 amino acids normalized with the total number of residues in a protein.

#### Dipeptide amino acid composition (DPC)

DPC^38^, Park and Kanehisa have proposed another sequence-based feature for classification of the protein sequences, there are 400 elements specify the number of occurrences of each amino acid dipeptide that normalized with the total number of dipeptides in a given protein sequence.

#### Composition of *k*-spaced amino acid pair (CKSAAP)

CKSAAP^39^ was employed, which depicts the frequencies of 400 types of amino acid pairs that are separated by *k* other amino acids within the peptide sequence, *k* = (1, 2, 3) are considered in this study.

#### Physicochemical properties (PCP)

The modlAMP package (v4.2.0, https://modlamp.org/)^40^ offers functions for calculating a variety of physicochemical properties on amino acid peptides, including sequence net charges, charge density, isoelectric point, aliphatic index, aromaticity, hydrophobic ratio, Boman index, and instability index. In this study, the net charge and charge density of peptides were observed depend on the pH of the surrounding medium. The aliphatic index is a measure of the thermal stability of peptides depend on the relative volume occupied by the aliphatic amino acids, A (Ala, alanine), I (Ile, isoleucine), L (Leu, leucine), and V (Val, valine)^22^. The aromaticity is the relative frequency of aromatic amino acids, F (Phe, phenylalanine), W (Trp, tryptophan), and Y (Tyr, tyrosine)^41^. The hydrophobic ratio is independent of the differential accumulation of hydrophobic and hydrophilic residues, the hydrophobic amino acids include A, C (Cys, cysteine), F, I, L, M (Met, methionine), and V^42^. The Boman index to measure the binding affinity of peptide-protein interactions, which is the sum of the free energies of the respective amino acid side chains divided by the total number of the residues in a peptide^24^. The instability index is a measure that predicts the in vivo stability of a protein based on the amino acid composition^23^.

### ACP subtypes classification based on the distribution of positively charged residues

In this study, we compared the sum of positive charge between the first (N-terminus) and the least (C-terminus) 5 amino acid residues for each peptide, then the training set was partitioned into three major groups depending on which area contains a higher concentration of positively charged residues such as H (His, histidine), K (Lys, lysine) and R (Arg, arginine). Thus, the peptides containing more positively charged residues around the C-terminus than N-terminus could be further divided into “group C+”, whereas having a higher concentration of positive charge around the N-terminus than C-terminus were split to the “group N+”, and the remaining data were classified into another group “group O”.

### Construction of the two-step machine learning models

The aim of this study is to develop a two-step machine learning model for identification of the subtypes of ACP, which classify the input data into the corresponding group before applying the classifier. The process for two-step machine learning models-building includes the following steps:

1. Data collection;
2. Sequence redundancy removal;
3. Subtype classification based on the distribution of positively charged residues;
4. Features investigation of each subtype independently;
5. Applying the corresponding classifier.

Based on the binary classification, support vector machine (SVM) is an advanced machine learning algorithm, which has been widely applied in the biological field. LIBSVM, a public SVM tool that adopt the radial basis function (RBF) as the kernel function, which determined by a gamma parameter (γ) while the cost parameter (C) was used to modulate the softness of the hyper-plane^43^. As described in a number of previous works^44–50^, using SVM with RBF kernel as the classifier is a reliable choice in protein function prediction; thus, in this study, LIBSVM was used to build the predictive models for discriminating the anticancer peptides from the peptides without anticancer activities. For each model training session, a grid search was performed for determining the penalty parameter C and the kernel parameter γ for SVM with RBF kernel using the grid.py script supplied by LIBSVM. The five-fold cross-validation was conducted for (C, γ), and then, the parameter combination leading to the highest accuracy was used to construct the prediction model.

### Five-fold cross validation and performance measurement

In order to avoid overfitting during the model training, the 5 repetitions of five-fold cross-validation procedure were conducted to estimate the performance of the models. For each group, the training dataset was randomly divided into five subsets of equal size, of which four sets are used for model training and the remaining set for validation. This procedure will be repeated five times, that is, until each of five subsets in the group serves as a testing set. The predicted results of five validation sets were then combined into a single performance, and then to estimate the predictive performance of the model, the following measures were used:

$$Sensitivity = \frac{{TP}}{{TP + FN}}$$

$$Specificity = \frac{{TN}}{{TN + FP}}$$

$$Accuracy = \frac{{TP + TN}}{{TP + FP + TN + FN}}$$

$$Balanced~Accuracy = \frac{{Sensitivity + Specificity}}{2}$$

$${\text{MCC}} = \frac{{\left( {TP \times TN} \right) - \left( {FP \times FN} \right)}}{{\sqrt {\left( {TP + FP} \right)\left( {TP + FN} \right)\left( {TN + FP} \right)\left( {TN + FN} \right)} }},$$

where true positive (TP) denotes the number of correctly labelled positive samples; false positive (FP) denotes the number of negative samples incorrectly labelled as positive; true negative (TN) denotes the number of correctly labelled negative samples; false negative (FN) denotes the number of positive samples incorrectly labelled as negative, and means and standard deviations are reported for all measures.

## Supplementary Information

Supplementary Tables.
