# Supplementary material for: Identification of subtypes of anticancer peptides based on sequential features and physicochemical properties
Source: Sci Rep. 2021 Jun 30;11:13594. doi: 10.1038/s41598-021-93124-9 (PMC8245499; doi:10.1038/s41598-021-93124-9)
Supplement: Supplementary file 1 — Supplementary Tables. [file 41598_2021_93124_MOESM1_ESM.docx]

**Identification of subtypes of anticancer peptides based on sequential features and physicochemical properties**

**Kai-Yao Huang**^1,2^**, Yi-Jhan Tseng**^1^**, Hui-Ju Kao**^1^**, Chia-Hung Chen**^1^**, Hsiao-Hsiang Yang**^1^ **and Shun-Long Weng**^2,3,4,^*

^1^Department of Medical Research, Hsinchu Mackay Memorial Hospital, Hsinchu City 300, Taiwan

^2^Department of Medicine, Mackay Medical College, New Taipei City 252, Taiwan

^3^Department of Obstetrics and Gynecology, Hsinchu Mackay Memorial Hospital, Hsinchu City 300, Taiwan

^4^Mackay Junior College of Medicine, Medicine, Nursing and Management College, Taipei City 112, Taiwan

^*^Corresponding authors: [4467@mmh.org.tw](mailto:4467@mmh.org.tw) (Shun-Long Weng)

**Supplementary Tables**

**Table S1. The 5 repetition of five-fold cross validation results of the models trained with single feature.**

| **Feature** | **Repetition** | **TP** | **FP** | **TN** | **FN** |
| --- | --- | --- | --- | --- | --- |
| AAC | 1 | 696 | 209 | 1391 | 104 |
|  | 2 | 686 | 208 | 1392 | 114 |
|  | 3 | 690 | 198 | 1402 | 110 |
|  | 4 | 692 | 203 | 1397 | 108 |
|  | 5 | 685 | 202 | 1398 | 115 |
| DPC | 1 | 683 | 237 | 1363 | 117 |
|  | 2 | 682 | 257 | 1343 | 118 |
|  | 3 | 684 | 251 | 1349 | 116 |
|  | 4 | 679 | 241 | 1359 | 121 |
|  | 5 | 687 | 244 | 1356 | 113 |
| CKSAAP, k=1 | 1 | 691 | 220 | 1380 | 109 |
|  | 2 | 681 | 223 | 1377 | 119 |
|  | 3 | 690 | 209 | 1391 | 110 |
|  | 4 | 691 | 215 | 1385 | 109 |
|  | 5 | 686 | 228 | 1372 | 114 |
| CKSAAP, k=2 | 1 | 686 | 229 | 1371 | 114 |
|  | 2 | 681 | 219 | 1381 | 119 |
|  | 3 | 683 | 212 | 1388 | 117 |
|  | 4 | 684 | 219 | 1381 | 116 |
|  | 5 | 686 | 214 | 1386 | 114 |
| CKSAAP, k=3 | 1 | 692 | 219 | 1381 | 108 |
|  | 2 | 694 | 211 | 1389 | 106 |
|  | 3 | 693 | 220 | 1380 | 107 |
|  | 4 | 694 | 212 | 1388 | 106 |
|  | 5 | 692 | 214 | 1386 | 108 |
| PCP | 1 | 571 | 461 | 1139 | 229 |
|  | 2 | 563 | 472 | 1128 | 237 |
|  | 3 | 573 | 469 | 1131 | 227 |
|  | 4 | 584 | 453 | 1147 | 216 |
|  | 5 | 570 | 461 | 1139 | 230 |

**Table S2. The 5 repetition of five-fold cross validation results of the two-step models trained with the single feature.**

| **Feature** | **Repetition** | **Group** | **TP** | **FP** | **TN** | **FN** |
| --- | --- | --- | --- | --- | --- | --- |
| AAC | 1 | C+ | 345 | 54 | 374 | 49 |
|  |  | N+ | 132 | 89 | 419 | 26 |
|  |  | O | 209 | 105 | 559 | 39 |
|  | 2 | C+ | 340 | 50 | 378 | 54 |
|  |  | N+ | 127 | 79 | 429 | 31 |
|  |  | O | 207 | 117 | 547 | 41 |
|  | 3 | C+ | 350 | 55 | 373 | 44 |
|  |  | N+ | 127 | 96 | 412 | 31 |
|  |  | O | 213 | 119 | 545 | 35 |
|  | 4 | C+ | 342 | 50 | 378 | 52 |
|  |  | N+ | 129 | 92 | 416 | 29 |
|  |  | O | 212 | 101 | 563 | 36 |
|  | 5 | C+ | 347 | 63 | 365 | 47 |
|  |  | N+ | 131 | 87 | 421 | 27 |
|  |  | O | 207 | 116 | 548 | 41 |
| DPC | 1 | C+ | 341 | 58 | 370 | 53 |
|  |  | N+ | 121 | 123 | 385 | 37 |
|  |  | O | 208 | 109 | 555 | 40 |
|  | 2 | C+ | 343 | 67 | 361 | 51 |
|  |  | N+ | 122 | 141 | 367 | 36 |
|  |  | O | 211 | 121 | 543 | 37 |
|  | 3 | C+ | 347 | 64 | 364 | 47 |
|  |  | N+ | 121 | 138 | 370 | 37 |
|  |  | O | 207 | 120 | 544 | 41 |
|  | 4 | C+ | 337 | 60 | 368 | 57 |
|  |  | N+ | 121 | 140 | 368 | 37 |
|  |  | O | 207 | 122 | 542 | 41 |
|  | 5 | C+ | 342 | 68 | 360 | 52 |
|  |  | N+ | 119 | 132 | 376 | 39 |
|  |  | O | 206 | 118 | 546 | 42 |
| CKSAAP, k=1 | 1 | C+ | 337 | 62 | 366 | 57 |
|  |  | N+ | 119 | 126 | 382 | 39 |
|  |  | O | 211 | 100 | 564 | 37 |
|  | 2 | C+ | 344 | 57 | 371 | 50 |
|  |  | N+ | 126 | 124 | 384 | 32 |
|  |  | O | 216 | 105 | 559 | 32 |
|  | 3 | C+ | 343 | 61 | 367 | 51 |
|  |  | N+ | 123 | 122 | 386 | 35 |
|  |  | O | 213 | 104 | 560 | 35 |
|  | 4 | C+ | 345 | 56 | 372 | 49 |
|  |  | N+ | 124 | 130 | 378 | 34 |
|  |  | O | 212 | 98 | 566 | 36 |
|  | 5 | C+ | 341 | 53 | 375 | 53 |
|  |  | N+ | 120 | 116 | 392 | 38 |
|  |  | O | 212 | 100 | 564 | 36 |
| CKSAAP, k=2 | 1 | C+ | 340 | 61 | 367 | 54 |
|  |  | N+ | 124 | 110 | 398 | 34 |
|  |  | O | 206 | 113 | 551 | 42 |
|  | 2 | C+ | 339 | 60 | 368 | 55 |
|  |  | N+ | 129 | 103 | 405 | 29 |
|  |  | O | 209 | 114 | 550 | 39 |
|  | 3 | C+ | 343 | 61 | 367 | 51 |
|  |  | N+ | 130 | 111 | 397 | 28 |
|  |  | O | 209 | 112 | 552 | 39 |
|  | 4 | C+ | 335 | 59 | 369 | 59 |
|  |  | N+ | 129 | 105 | 403 | 29 |
|  |  | O | 204 | 109 | 555 | 44 |
|  | 5 | C+ | 338 | 62 | 366 | 56 |
|  |  | N+ | 124 | 104 | 404 | 34 |
|  |  | O | 199 | 125 | 539 | 49 |
| CKSAAP, k=3 | 1 | C+ | 346 | 53 | 375 | 48 |
|  |  | N+ | 121 | 120 | 388 | 37 |
|  |  | O | 208 | 110 | 554 | 40 |
|  | 2 | C+ | 344 | 50 | 378 | 50 |
|  |  | N+ | 128 | 126 | 382 | 30 |
|  |  | O | 209 | 112 | 552 | 39 |
|  | 3 | C+ | 344 | 50 | 378 | 50 |
|  |  | N+ | 127 | 129 | 379 | 31 |
|  |  | O | 206 | 104 | 560 | 42 |
|  | 4 | C+ | 341 | 49 | 379 | 53 |
|  |  | N+ | 125 | 116 | 392 | 33 |
|  |  | O | 205 | 92 | 572 | 43 |
|  | 5 | C+ | 343 | 51 | 377 | 51 |
|  |  | N+ | 134 | 116 | 392 | 24 |
|  |  | O | 209 | 108 | 556 | 39 |
| PCP | 1 | C+ | 301 | 107 | 321 | 93 |
|  |  | N+ | 114 | 143 | 365 | 44 |
|  |  | O | 165 | 226 | 438 | 83 |
|  | 2 | C+ | 294 | 108 | 320 | 100 |
|  |  | N+ | 111 | 168 | 340 | 47 |
|  |  | O | 169 | 225 | 439 | 79 |
|  | 3 | C+ | 298 | 98 | 330 | 96 |
|  |  | N+ | 112 | 141 | 367 | 46 |
|  |  | O | 199 | 305 | 359 | 49 |
|  | 4 | C+ | 301 | 98 | 330 | 93 |
|  |  | N+ | 113 | 137 | 371 | 45 |
|  |  | O | 167 | 237 | 427 | 81 |
|  | 5 | C+ | 304 | 118 | 310 | 90 |
|  |  | N+ | 116 | 148 | 360 | 42 |
|  |  | O | 178 | 201 | 463 | 70 |

**Table S3. The 5 repetition of five-fold cross validation results of the two-step models trained with the hybrid feature sets**

| **Feature** | **Repeat** | **Group** | **TP** | **FP** | **TN** | **FN** |
| --- | --- | --- | --- | --- | --- | --- |
| AAC + DPC | 1 | C+ | 347 | 52 | 376 | 47 |
|  |  | N+ | 131 | 91 | 417 | 27 |
|  |  | O | 215 | 89 | 575 | 33 |
|  | 2 | C+ | 344 | 49 | 379 | 50 |
|  |  | N+ | 133 | 90 | 418 | 25 |
|  |  | O | 213 | 89 | 575 | 35 |
|  | 3 | C+ | 349 | 50 | 378 | 45 |
|  |  | N+ | 127 | 90 | 418 | 31 |
|  |  | O | 212 | 92 | 572 | 36 |
|  | 4 | C+ | 343 | 47 | 381 | 51 |
|  |  | N+ | 130 | 93 | 415 | 28 |
|  |  | O | 210 | 93 | 571 | 38 |
|  | 5 | C+ | 339 | 52 | 376 | 55 |
|  |  | N+ | 133 | 93 | 415 | 25 |
|  |  | O | 208 | 94 | 570 | 40 |
| AAC + DPC  + PCP | 1 | C+ | 348 | 52 | 376 | 46 |
|  |  | N+ | 131 | 87 | 421 | 27 |
|  |  | O | 215 | 89 | 575 | 33 |
|  | 2 | C+ | 348 | 52 | 376 | 46 |
|  |  | N+ | 130 | 82 | 426 | 28 |
|  |  | O | 213 | 92 | 572 | 35 |
|  | 3 | C+ | 348 | 46 | 382 | 46 |
|  |  | N+ | 130 | 88 | 420 | 28 |
|  |  | O | 213 | 95 | 569 | 35 |
|  | 4 | C+ | 345 | 44 | 384 | 49 |
|  |  | N+ | 129 | 88 | 420 | 29 |
|  |  | O | 209 | 90 | 574 | 39 |
|  | 5 | C+ | 345 | 53 | 375 | 49 |
|  |  | N+ | 129 | 85 | 423 | 29 |
|  |  | O | 209 | 95 | 569 | 39 |
| AAC + DPC  + CKSAAP | 1 | C+ | 343 | 57 | 371 | 51 |
|  |  | N+ | 127 | 106 | 402 | 31 |
|  |  | O | 213 | 94 | 570 | 35 |
|  | 2 | C+ | 349 | 55 | 373 | 45 |
|  |  | N+ | 131 | 106 | 402 | 27 |
|  |  | O | 209 | 88 | 576 | 39 |
|  | 3 | C+ | 353 | 55 | 373 | 41 |
|  |  | N+ | 128 | 109 | 399 | 30 |
|  |  | O | 212 | 90 | 574 | 36 |
|  | 4 | C+ | 349 | 52 | 376 | 45 |
|  |  | N+ | 132 | 103 | 405 | 26 |
|  |  | O | 209 | 90 | 574 | 39 |
|  | 5 | C+ | 350 | 57 | 371 | 44 |
|  |  | N+ | 130 | 106 | 402 | 28 |
|  |  | O | 212 | 90 | 574 | 36 |
| AAC + DPC  + CKSAAP + PCP | 1 | C+ | 345 | 54 | 374 | 49 |
|  |  | N+ | 127 | 100 | 408 | 31 |
|  |  | O | 214 | 92 | 572 | 34 |
|  | 2 | C+ | 347 | 52 | 376 | 47 |
|  |  | N+ | 129 | 102 | 406 | 29 |
|  |  | O | 207 | 91 | 573 | 41 |
|  | 3 | C+ | 351 | 49 | 379 | 43 |
|  |  | N+ | 129 | 103 | 405 | 29 |
|  |  | O | 212 | 95 | 569 | 36 |
|  | 4 | C+ | 349 | 47 | 381 | 45 |
|  |  | N+ | 135 | 96 | 412 | 23 |
|  |  | O | 210 | 93 | 571 | 38 |
|  | 5 | C+ | 348 | 53 | 375 | 46 |
|  |  | N+ | 127 | 100 | 408 | 31 |
|  |  | O | 211 | 90 | 574 | 37 |

**Table S4. Comparison of independent testing results between our method and the available prediction tools.**

| **Group** | **TP** | **FP** | **TN** | **FN** | **Sen.** | **Spec.** | **Acc.** | **BAcc.** | **MCC** |
| --- | --- | --- | --- | --- | --- | --- | --- | --- | --- |
| C+ | 78 | 8 | 95 | 16 | 77.60% | 94.74% | 88.99% | 86.17 | 0.749 |
| N+ | 25 | 8 | 123 | 14 |  |  |  |  |  |
| O | 46 | 4 | 142 | 13 |  |  |  |  |  |

*BAcc.: balanced accuracy; MCC: Matthews correlation coefficient.
